# Factors influencing health-promoting behavior among single mothers in Northeastern Malaysia: a cross-sectional study

**DOI:** 10.7717/peerj.18359

**Published:** 2024-10-31

**Authors:** Saidah Adilah Mohamed Yusof, Tengku Alina Tengku Ismail, Kamarul Imran Musa, Hasmaryanti Kamaruzzaman

**Affiliations:** 1 Department of Community Medicine, School of Medical Sciences, Health Campus, Universiti Sains Malaysia, Kubang Kerian, Kelantan, Malaysia; 2 Women Development Department State of Kelantan, Pengkalan Chepa, Kelantan, Malaysia

**Keywords:** Health-promoting behavior, Health beliefs, Social support, Cardiovascular disease, Single mothers

## Abstract

**Introduction:**

Health-promoting behaviors (HPB) play a vital role in maintaining and enhancing overall well-being. Single mothers are at higher risk of cardiovascular disease (CVD) and less involvement in HPB due to psychosocial disadvantages.

**Objective:**

This study aimed to determine the HPB scores and factors influencing HPB among single mothers in Kelantan (Northeastern, Malaysia).

**Methods:**

This study employed a cross-sectional design, selecting 242 single mothers from Kelantan through proportional stratified sampling. Data were gathered through questionnaires covering sociodemographic details, the Health-Promoting Lifestyle Profile-II (HPLP-II), the Malay Version of Health Beliefs Related to Cardiovascular Disease (HBCVD-M), and Multidimensional Perceived Social Support (MPSS). The relationships between the dependent variable (HPB) and independent variables were analyzed using multivariable linear regression models.

**Results:**

The respondents achieved an average HPB score of 118.03 (SD = 19.2), with the highest mean scores in spiritual growth (22.46 [SD = 3.70]) and interpersonal relationships (22.05 [SD = 3.67]). Physical activity had the lowest mean score at 15.09 (SD = 4.62). Significant positive associations were found between HPB and perceived severity of CVD (adjusted β = 1.60; 95% CI [0.68–2.53]; *p* < 0.001) as well as perceived social support (adjusted β = 0.63; 95% CI [0.37–0.90]; *p* < 0.001). Conversely, educational level (adjusted β = −10.36; 95% CI [−16.06 to −4.67]; *p* < 0.001) and perceived benefits of reducing CVD risk (adjusted β = −1.43; 95% CI [−2.37 to −0.48]; *p* < 0.001) were negatively associated with HPB.

**Conclusions:**

The findings highlight the importance of health beliefs, social support, and education in shaping HPB among single mothers. Community health initiatives targeting this population should develop strategies to strengthen individuals’ health beliefs and promote a supportive environment.

## Introduction

Health-promoting behaviors (HPBs) are activities that improve self-realization and well-being, assisting individuals in maintaining and developing healthy lives (Pender, Murdaugh & Parsons, 2013). The goal of HPBs is to maximize well-being, achieve personal fulfilment, and lead productive lives, reflecting the human desire for self-actualization (Pender, Murdaugh & Parsons, 2013). HPBs include self-initiated behaviors such as eating a healthy diet, keeping an adequate body weight, exercising frequently, obtaining enough sleep, quitting smoking, and limiting alcohol consumption (Luyster et al., 2012). Participating in these actions protects against and lowers chronic diseases such as diabetes, obesity, cardiovascular disease (CVD) and cerebrovascular disease (Kholoud, 2020).

Age, gender, education, employment status, family income, perceived social support, self-efficacy, self-esteem, and prior health behaviors are some factors affecting HPBs. Other factors include health knowledge, marital status, and health beliefs (perceived benefit, perceived barriers, and perceived susceptibility) (Baheiraei et al., 2013; Anbari, Mostafavi & Ghanadi, 2014; Sehhatie, Mirghafourvand & Momeni, 2015; Chen et al., 2019; Lim, Noh & Kim, 2015; Shaheen Abeer et al., 2015; Mirghafourvand et al., 2013). Health beliefs in disease preventability significantly impact health behaviors. Therefore, evaluating one’s health beliefs is crucial, as attitudes and beliefs significantly influence health behaviors. Taiwanese research indicates that perceived susceptibility, perceived benefits, and perceived barriers are positively linked with stress management (SM) and interpersonal relationships (IR) (Chen et al., 2019). Furthermore, adopting healthy behaviors and a healthy lifestyle is influenced by individual characteristics and socioenvironmental factors (Kazemi & Hajian, 2018). Social support is a critical factor in coping with health problems and protecting against the harmful effects of stress (Nasrin et al., 2021). Social support significantly correlates with HPB, with a one-point improvement in social support scores leading to a 40% rise in HPB (Nasrin et al., 2021).

Pender categorized HPBs into six areas: stress management, physical activity, interpersonal relationships, spiritual growth, health responsibility, and nutrition (Pender, Murdaugh & Parsons, 2013). Single mothers, a socially and economically vulnerable group, face various physical and mental health problems (Amiri et al., 2014; Liang, Berge & Brand, 2019). In Malaysia the number of single mothers is increasing in trends (DOSM Department of Statistics Malaysia, 2023). Adopting HPBs is challenging for single mothers due to economic and social issues (Dor, 2021). Local studies revealed that single mothers face stressful psychological, physical and emotional needs alone while concurrently attending to their children’s needs (Hamid & Salleh, 2013). Single mothers report difficulties with physical activity, including lack of spare income, being sole caregivers, and lack of social and community support for physical activity (Dlugonski et al., 2017). Psychological concerns, conflicts between work and family responsibilities, financial constraints, and the parenting process were among the challenges reported by local researchers (Hamid & Salleh, 2013; Jusoh & Latada, 2020; Mulia, 2020). Additionally, single mothers face nutritional issues like low fruit and vegetable consumption due to social disadvantages (Sakinah et al., 2017). Given the challenges of HPBs among single mothers, this study aims to determine the average HPB scores among single mothers in Kelantan and to examine the relationships between sociodemographic factors, clinical profiles, health beliefs, perceived social support, with HPB. The study hypothesized that there are significant relationships between these variables with HPB scores among single mothers in Kelantan.

## Materials & Methods

### Study design and respondents

This cross-sectional study was conducted from November 2023 until April 2024 in four randomly selected districts in Kelantan, one of the states located in East Coast Malaysia. Inclusion criteria were single mothers aged 18 to 60 registered under the Women Development Departments of Kelantan with no underlying CVD. Non-Malaysians were excluded from the study. The sample size was determined based on the objective of the study. To determine the average HPB scores, the sample size was calculated using the web calculator by Arifin (2013) and Naing (2003). The calculated sample size was 242 after considering a 20% dropout rate. Meanwhile, to determine the relationship, the sample size was calculated based on G power version 3.1.9.7 Software. The effect size was set at medium Cohen of 0.15 and 15 predictors for multiple linear regression statistical analysis (Faul et al., 2009). The total sample size obtained was 172. Therefore, the biggest sample size of the study was 242, which was derived from the calculation in the first objective, was selected as the total sample size of the study. A stratified sampling proportionate to size was performed to obtain the required sample size from each selected district. Later, the respondents from each district were recruited by simple random sampling.

## Research tools

Data was collected using the following tools:

First, a proforma was utilized, with questions about the respondents’ clinical profiles (underlying comorbidities) and demographics (age, education level, employment status, marital status, household income and ethnicity) included. Comorbidity was noted when respondents reported existing diseases, such as diabetes, hypertension, kidney failure, asthma, *etc*.

The second tool was the Health Beliefs Related to Cardiovascular Disease (HBCVD) Scale’s Malay adaptation (HBCVD-M), developed by Tovar et al. (2010). The four subscales of the health beliefs questionnaire are perceived barriers, perceived benefits, perceived severity, and perceived susceptibility. This scale contains 25 items. For every item, there were four possible responses ranging from strongly disagree (1) to strongly agree (4). The higher score indicates a stronger belief. The total score for each subscale was calculated. The HBCVD-M has good model fit indices: standardized root means square residual (SRMR) = 0.060; Tucker–Lewis index (TLI) = 0.90; confirmatory factor index (CFI) = 0.92, Root means square error of approximation (RMSEA) = 0.072 (90 CI% [0.061–0.082]); and reported good internal consistency reliability with composite reliability (Raykov’s Rho) ranging from 0.745 to 0.902 and Cronbach’s alpha of 0.744 to 0.890 (Mohamed Yusof et al., 2023).

The third tool was the Multidimensional Perceived Social Support (MPSS) questionnaire, which was translated and validated into Malay (Ng et al., 2010). Zimet Gregory et al. (1990) created the MPSS questionnaire in 1988. It has 12 items in total among its three subscales: family support, friend support, and significant other support. Very strongly disagree (1) to very strongly agree (7) were the range of response selections. The sum of the scores was determined. The lower score indicates poor social support. MPSS questionnaires have been validated in Malaysia among medical students (Ng et al., 2010) and teachers (Cheng, Foong & Naqiah, 2017). The instrument has strong internal consistency with Cronbach alpha = 0.89, parallel form reliability (0.94) and test-retest reliability (0.77) (Spearman’s rho, *p* < 0.01) (Ng et al., 2010).

Lastly, the fourth tool employed was the validated and translated Malay version of Health Promoting Lifestyle Profile II (HPLP II) to evaluate the HPB of the respondents. HPLP II questionnaire was developed by Pender et al. in 1987 based on Pender’s health promotion model (Walker, Sechrist & Pender Nola, 1987). It consists of six subscales as follows: interpersonal relationship (nine items), health responsibility (nine items), physical activity (eight items), spiritual growth (nine items), nutrition (nine items), and stress management (eight items). The questionnaire used a 4-point Likert scale, from 1 = “never”, 2 = “sometimes”, 3 = “often” and 4 = “routinely”. The total score was calculated for each subscale for comparison across subscales. A higher mean total score indicates higher tendencies to engage in HPB. This questionnaire has been translated and validated into Malay by Kuan et al. (2019). Confirmatory factor analysis (CFA) was conducted among university students. The reported Cronbach alpha for the overall scale is 0.94, while the Cronbach alpha range for each subscale is 0.730 to 0.87. The fit indices are RMSEA = 0.046, SRMR = 0.062, CFI = 0.814 to 0.821, and TLI = 0.805 to 0.811. The overall factor loadings for each item are more than 0.4. The goodness-of-fit indices were poor in accordance with the Hu and Bentler criteria: Tucker–Lewis index (TLI) of ≥0.95, comparative fit index (CFI) of ≥0.95, and root mean square error of approximation (RMSEA) of ≤0.06 as cited by Lim et al. (2021).

### Data collection

Data collection was done in cooperation with the Women’s Development Department of State of Kelantan (WDD). Every month, the WDD schedules a few community-based programs for women such as cooking demonstration classes, sewing classes, craft creative programs such as flower arrangement, surprise gift decoration and arrangement. Selected participants were approached individually during the program. The study was explained, and informed written consent was obtained. Participants completed the self-administered questionnaire, which was checked for completeness. Participant privacy and data confidentiality were maintained.

### Ethical approval

The Human Research Ethics Committee of Universiti Sains Malaysia (USM/ JEPeM/KK/ 23010110) granted ethical permission for this work, and it was registered with the National Medical Research Register (NMRR-23-00487-DCY).

### Statistical analysis

Data entry from the questionnaire booklets was performed using Microsoft Excel and exported to RStudio Version: 2023.12.1+402 IDE. Preliminary data screening identified and rectified missing values and entry errors. Descriptive statistics summarized respondents’ socio-demographic features, health beliefs on CVD, perceived social support, and HPB items. Numerical data were presented as mean and standard deviation (SD), and categorical data as frequency and percentage (%). Simple and multiple linear regression analyses determined associations between independent variables (socio-demographic, clinical profiles, health beliefs, and perceived social support) and the dependent variable (total HPB score). An α of 0.05 was set for statistical significance. Only variables with a *p*-value < 0.25 in univariable analysis were included in multivariable analysis.

## Results

### Socio-demographic and clinical characteristics of single mothers

A total of 242 single mothers participated, with a mean age of 47.9 (8.72) years. Most respondents were Malay (92.6%) and widows (62%). Over 80% had primary and secondary education (80.2%), and the majority had a household income of less than RM3030 (93.4%). The details of these findings can be found in Table 1.

**Table 1:** Socio-demographic and clinical characteristics of single mothers.

**Variables**	**n (%)**	**Mean (SD)**
Age (year)		47.9 (8.72)
Race		
Malay	224 (92.6)	
Chinese	14 (5.8)	
Indian	4 (1.6)	
Marital status		
Widow	150 (62.0)	
Divorcee	92 (38.0)	
Highest formal education		
No formal education	6 (2.4)	
Primary & Secondary school	194 (80.2)	
Certificate/Diploma/STPM	29 (12.0)	
Degree	12 (5.0)	
Master/Phd	1 (0.4)	
Employment Status		
Unemployed	136 (56.2)	
Employed	106 (43.8)	
Household Income		
<RM3030 (B40) [<$698.56 usd]	226 (93.4)	
RM3030 to RM6619 (M40) [$698.56 to $1 529,35 usd]	12 (5.0)	
≥RM6620(T20) [> $1 529,58 usd]	4 (1.6)	
Comorbidities		
Yes	94 (38.8)	
No	148 (61.2)	

**Notes.**

n, number of respondents; SD, standard deviation.

### Health beliefs related to CVD among single mothers

Table 2 displays the subscale scores for each construct of the HBCVD scales. The mean scores for perceived susceptibility, perceived severity, perceived benefits, and perceived barriers were 11.01 (3.56), 13.73 (2.32), 10.01 (2.33), and 10.83 (2.34), respectively.

**Table 2:** Health beliefs related to CVD among single mothers.

**Construct (no of items)**	**Min–Max**	**Mean** ^a^	**SD**
Perceived susceptibility (5)	5, 20	11.01	3.56
Perceived severity (5)	5, 20	13.73	2.32
Perceived benefits (6)	6, 13	10.01	2.33
Perceived barriers (5)	5, 17	10.83	2.34
Total score	31, 61	45.6	5.92

**Notes.**

a Mean obtained from the total score

SD, standard deviation.

### Perceived social support among single mothers

Perceived social support consisted of three constructs: significant others, family and friends. The mean scores of each construct range from 18.29 (4.77) to 20.62 (5.22). Details of the score are presented in Table 3.

**Table 3:** Perceived social support among single mothers.

**Construct (no of items)**	**Min–Max**	**Mean** ^a^	**SD**
Significant others (4)	4, 28	19.10	5.36
Family (4)	4, 28	20.62	5.22
Friends (4)	4, 28	18.29	4.77
Total score	12, 84	60.17	8.14

**Notes.**

a Mean obtained from the total score.

SD, standard deviation.

### Mean scores of HPB subscales among single mothers

Notably, the spiritual growth subscale recorded the highest score, with a mean of 22.46 (3.70). Conversely, physical activity obtained the lowest score, with a mean of 15.09 (4.62), followed by health responsibility, which had a mean of 19.06 (4.65). The comprehensive results are outlined in the associated Table 4.

**Table 4:** Mean scores of HPB subscales among single mothers.

**Construct (items)**	**Min, Max**	**Mean (SD)** ^a^
Health responsibility (9)	10, 34	19.06 (4.65)
Physical activity (8)	8, 30	15.09 (4.62)
Nutrition (9)	13, 31	20.52 (3.42)
Spiritual growth (9)	14, 33	22.46 (3.70)
Interpersonal relations (9)	13, 32	22.05 (3.67)
Stress management (8)	11, 27	19.72 (3.47)
Total score of HPB	70, 170	118.03 (19.2)

**Notes.**

a Mean of raw score.

SD, standard deviation.

### Influence of sociodemographic characteristics, clinical profiles, health beliefs related to CVD and perceived social support with total HPB scores

Univariable analysis identified seven variables with *p* < 0.25: age, education, household income, perceived severity, benefits, barriers, and social support. In the multivariate model, education, perceived severity, benefits, and social support remained significant (*p* < 0.05). Secondary education or lower was associated with decreased HPB scores by 10.36 units. The perceived severity of CVD increased HPB scores by 1.60 units per unit increase. Perceived benefits are inversely related to HPB scores, decreasing by 1.43 units per unit increase. Higher perceived social support increased HPB scores by 0.63 units per unit increase. The model explained 23.6% of the variation in HPB scores as presented in Table 5.

**Table 5:** Relationships between sociodemographic characteristics, clinical profiles, health beliefs related to CVD and perceived social support with total HPB scores among single mothers in Kelantan (*n* = 242).

**Variables**	**Simple linear regression**		**Multiple linear regression**	
	**Crude** ***b*** ^a^ **(95% CI)**	***p*-value**	**Adjusted** ***b*** ^b^ **(95% CI)**	***p*-value**
Age	0.27 (−0.004, 0.55)	0.054		
Race				
Malay	0			
Non-Malay	−3.16 (−12.42, 6.11)	0.503		
Marital status				
Divorcee	0			
Widow	−0.44 (−5.45, 4.57)	0.864		
Highest formal education				
Tertiary & upper	0			
Secondary & lower	−13.70 (19.89, 7.51)	<0.001	−10.36 (16.06, 4.67)	<0.001
Employment status				
Employed	0			
Unemployed	−0.14 (−5.05, 4.77)	0.954		
Household income				
>RM3030 (M40 & T20)	0			
<RM3030(B40)	−18.30 (−28.13, 8.47)	<0.001		
Comorbidities				
No	0			
Yes	0.90 (−4.09, 5.89)	0.722		
Health beliefs related to CVD				
Perceived susceptibility	0.21 (−0.48, 0.89)	0.549		
Perceived severity	2.14 (1.12, 3.15)	<0.001	1.60 (0.68, 2.53)	<0.001
Perceived benefits	−2.36 (−3.36, −1.36)	<0.001	−1.43 (−2.37, −0.48)	0.003
Perceived barriers	−1.15 (−2.18, −0.11)	0.029		
Perceived social support	0.82 (0.54, 1.09)	<0.001	0.63 (0.37, 0.90)	<0.001

**Notes.**

a Crude regression coefficient

b Adjusted regression coefficient (*R*^2^ = 0.236)

Forward multiple linear regression method applied. Model assumptions are fulfilled.

No interactions between independent variables were identified. No multi-collinearity

## Discussion

In this study, the total score of HPB was 118.03 ± 19.2, which was comparatively lower than values reported in other studies conducted in diverse countries among various target populations. For instance, in studies on urban Chinese women, 123.53 ± 20.27 (Cheng et al., 2015), middle-aged women in Iran, 124.42 ± 19.18 (Amirabadizadeh, Sharifzadeh & Moodi, 2016), and pregnant women in Turkey, 130.7 ± 20.0 (Onat & Aba, 2014) reported higher total scores. Conversely, the total scores obtained in this study were higher than previous research among women of reproductive age in Iran, 106.64 ± 11.93 (Shaahmadi et al., 2019).

The discrepancies in findings may be attributed to cultural and socioeconomic differences. Cultural norms, beliefs, and socioeconomic factors play pivotal roles in influencing health behaviors (Kyonne, 2019; Kraft & Kraft, 2021). For example, perceptions of healthy behavior may vary across cultures, leading to differences in adherence to health-promoting activities. Additionally, socioeconomic status can impact access to healthcare resources, knowledge about health behaviors, and the ability to engage in certain health-promoting activities.

In this study, single mothers scored highest in spiritual growth (22.46 ± 3.70), followed by interpersonal relations (22.05 ± 3.67), a trend consistent with findings from previous studies (Cheng et al., 2015; Onat & Aba, 2014; Mirghafourvand et al., 2014). However, other studies reported contrary results (Amirabadizadeh, Sharifzadeh & Moodi, 2016; Shaahmadi et al., 2019). Spiritual growth reflects individuals’ potential to fulfil themselves and strive to be their best selves. This encompasses expressions of creativity, the pursuit of spiritual enlightenment, the thirst for knowledge, and the desire to contribute to society (Cheng et al., 2015). Given the emotional and mental challenges often experienced in single motherhood, many single mothers turn to spiritual practices or religious beliefs for solace, strength, and guidance. Spirituality can offer a sense of purpose, meaning, and inner peace, which is particularly valuable when navigating the unique difficulties of single parenthood.

The strong scores in spiritual growth and interpersonal relationships align with traditional Malay cultural values, where spirituality and community ties play a central role in daily life. Previous studies have highlighted the role of spiritual and social support in promoting well-being, particularly in communities with strong religious and cultural practices, such as the Malay community (Dai et al., 2022; Kruk & Aboul-enein, 2024).

Single mothers in our study scored lowest in physical activity, a trend consistent with findings from various studies examining health behaviors across different groups (Cheng et al., 2015; Amirabadizadeh, Sharifzadeh & Moodi, 2016; Onat & Aba, 2014; Shaahmadi et al., 2019). Moreover, several studies have highlighted higher levels of physical inactivity among postmenopausal women (Sehhatie, Mirghafourvand & Momeni, 2015; Moshfeghy et al., 2023; Rahimi et al., 2023). This may have influenced our study’s findings, given that the average age of single mothers was 47.9 years old, close to menopause. According to the most recent local guideline on menopause, the average age of menopause among Malaysian women is 50.7 years.

Our findings suggest that single mothers lead sedentary lifestyles, a prevalent challenge observed in many countries. Such sedentary behavior poses a significant risk factor for various diseases and their chronic complications, including CVD. Several factors may contribute to this low level of physical activity among single mothers. These include inadequate exercise facilities within the community, lack of social support or a physical activity partner, time constraints, limited knowledge about exercise, inadequate safe outdoor play areas, low self-efficacy regarding exercise, and the absence of walking tracks, particularly for females, in most cities (Rahimi et al., 2023).

Study findings revealed that education level was the sole sociodemographic factor influencing HPB. Respondents with secondary education or lower demonstrated significantly lower HPB scores than those with tertiary education or higher (β = −10.36, *p* < 0.001). Our findings align with previous studies conducted among women of reproductive age (Bakouei et al., 2017). In a different study on HPB in patients with CVD, education level was also found to be a significant predictor of HPB (β = 0.198, *p* = 0.031), along with age (β = 0.247, *p* = 0.015) and the existence of diabetes (β = 0.166, *p* = 0.032) (Wicaksana et al., 2021). Moreover, Gokyildiz et al. (2014) found that participants’ levels of education were correlated with the degree to which they engaged in lifestyle practices that promote health.

For the health beliefs related to CVD, only perceived severity related to CVD (β = 1.60, *p* < 0.001) and perceived benefits in actions that reduced the risk of CVD (β = −1.43, *p* = 0.003) remained significantly related to the total scores of HPB among single mothers. This finding is consistent with previous research examining factors affecting HPB in young, elderly adults, where predictors included previous health-related behaviors (β = 0.154, *p* = 0.033) and perceived benefit (β = 0.738, *p* < 0.001), accounting for 66.2% of the variance in HPB (Lim, Noh & Kim, 2015). Similarly, a study among pregnant women identified perceived benefit as a predictor for HPB (β = 0.63, *p* < 0.001) (Bahabadi et al., 2020). However, contradicting results were obtained from a study involving female Iranian households, where no significant relation was found between perceived benefit and HPB (Khosravan et al., 2019).

Our research found no significant association between perceived susceptibility and perceived barriers to CVD with HPB. This finding may be attributed to competing priorities and time constraints. Single mothers often juggle multiple responsibilities, including childcare, household duties, and possibly employment (Dor, 2021).

In this study, social support demonstrated a significant relationship with HPB (β = 0.63, *p* < 0.001). This finding aligns with recent research (Kholoud, 2020; Nasrin et al., 2021; Khosravan et al., 2019). Taechaboonsermsak et al. (2020) found that social support directly influences health and well-being. However, the relationship between social support and HPB in women remains complex and relatively understudied (Mirghafourvand et al., 2014). While some studies have shown no association between social support and HPB, as reported by Shaahmadi et al. (2019).

Given the importance of social support and spiritual growth observed in this study, future interventions could focus on leveraging these cultural strengths. Community-based programs that integrate spiritual guidance and enhance social support networks may help single mothers adopt healthier behaviors. Health campaigns could collaborate with local religious and community leaders to encourage physical activity and healthy nutrition in ways that resonate with Malay cultural values.

Empirical research on the determinants of HPB among single mothers, particularly in Malaysia, is limited. Conducted based on extensive literature reviews and supported by theory-based investigation, this study addresses this gap by examining the relationships between health beliefs, social support, and HPB. The results contribute to narrowing the important gap in the literature, especially concerning HPB among single mothers, by identifying key determinants. A potential weakness of this study is that the self-report data collection approach may have increased or decreased participant inclination to overestimate their HPB. The cross-sectional nature of the investigation constituted another constraint. It is, therefore, impossible to consider the correlations between the HPBs and the associated factors as causative. It is advisable to consider the significance of HPBs and assess them in different age groups. To fully understand women’s perspectives and experiences with HPBs, additional qualitative study on the impact of other factors is also required.

## Conclusions

The present study underscores the noteworthy influence of health beliefs, sociodemographic characteristics, and social support on the formation of HPB among single mothers in Kelantan. With the greatest scores in spiritual development and interpersonal relationships and the lowest in physical activity, the individuals’ overall HPB score was modest. While lower education level and perceived benefits were inversely linked with HPBs, perceived social support and the severity of cardiovascular illness were positively associated with higher HPB scores.

These findings highlight the necessity of including cultural and social factors into health promotion programs. Given this population’s strong reliance on spirituality and interpersonal ties, community health interventions should prioritize improving social support networks and creating conditions that promote active lives. Targeted treatments, particularly those that take into account educational backgrounds, have the potential to boost participation in health-promoting activities. More study is needed to investigate additional cultural elements and how they may influence health behaviors among single mothers, resulting in a more comprehensive understanding of the interventions required to promote healthier lifestyles in this population.

## Supplemental Information

10.7717/peerj.18359/supp-1 Supplemental Information 1 Dataset The demographic characteristics of the respondents, together with the scores obtained for the three tools incorporated in the study: Health beliefs related to cardiovascular disease, multidimensional perceived social support, and health-promoting lifestyle profile II.

## Additional Information and Declarations

### Data Availability

The following information was supplied regarding data availability: The raw data is available in the Supplementary File.
